# Diagnostic Challenges in Pediatric Fever of Unknown Origin: Combined Role of Ferritin and Fever Duration

**DOI:** 10.3390/children12111493

**Published:** 2025-11-04

**Authors:** Pınar Önal, Gözde Apaydın Sever, Beste Akdeniz Eren, Gülşen Kes, Esra Özek Yücel, Süheyla Ocak, Ayça Kıykım, Sezgin Şahin, Haluk Çokuğraş, Fatih Aygün, Özgür Kasapçopur, Fatma Deniz Aygün

**Affiliations:** 1 Department of Pediatric Infectious Diseases, Cerrahpaşa Faculty of Medicine, Istanbul University-Cerrahpasa, İstanbul 34098, Turkey; pinar.onal@iuc.edu.tr (P.Ö.); gozde_apaydin@hotmail.com (G.A.S.); besteak.90@gmail.com (B.A.E.); gulsen.kes.17@gmail.com (G.K.); halukcezmi.cokugras@iuc.edu.tr (H.Ç.); 2 Department of Pediatric Allergy Immunology, Cerrahpaşa Faculty of Medicine, Istanbul University-Cerrahpasa, İstanbul 34098, Turkey; esra.yucel@iuc.edu.tr (E.Ö.Y.); ayca.kiykim@iuc.edu.tr (A.K.); 3 Department of Pediatric Hematology Oncology, Cerrahpaşa Faculty of Medicine, Istanbul University-Cerrahpasa, İstanbul 34098, Turkey; suheylaocak@iuc.edu.tr; 4 Department of Pediatric Rheumatology, Cerrahpaşa Faculty of Medicine, Istanbul University-Cerrahpasa, İstanbul 34098, Turkey; sezgin.sahin@iuc.edu.tr (S.Ş.); ozgurkc@iuc.edu.tr (Ö.K.); 5 Department of Pediatric Intensive Care, Cerrahpaşa Faculty of Medicine, Istanbul University-Cerrahpasa, İstanbul 34098, Turkey; fatih.aygun@iuc.edu.tr

**Keywords:** fever of unknown origin, children, ferritin, infectious, fever duration

## Abstract

**Highlights:**

**What are the main findings?**

- Infectious and rheumatologic diseases are the primary causes for fever of unknown origin in children, while a notable subset of undefined cases resolves spontaneously without recurrence.
- Prolonged fever combined with elevated ferritin emerged as a combined predictor of non-infectious etiologies.

**What is the implication of the main finding?**

- A simple combination of prolonged fever duration and ferritin can provide an early, practical tool to differentiate non-infectious etiologies.

**Abstract:**

**Background:** Fever of unknown origin (FUO) in children remains a diagnostic challenge due to heterogeneous etiologies. This study investigated the etiological distribution, long-term outcomes of undefined cases, and laboratory predictors that differentiate infectious from non-infectious etiologies. **Methods**: We retrospectively evaluated 87 children (1 month–18 years) hospitalized with fever > 38.3 °C for ≥7 days with no detectable source (2018–2024). Patients were categorized into five groups: infectious, inflammatory, neoplastic, miscellaneous, and undefined. Comparisons between these groups were performed in terms of age, laboratory values, and duration of fever using the Kruskal–Wallis test and one-way ANOVA. Demographic, clinical, laboratory, and follow-up data were compared. ROC analysis and binary logistic regression identified predictors of non-infectious etiologies. **Results**: Infectious diseases (42.5%) and inflammatory disorders (19.5%) were the most common causes, while 17.2% of cases remained undefined. The median age was 60 months. Rash (31%) and fatigue (27.5%) were the most common complaints on admission. The undefined group showed complete spontaneous resolution during a median 63-month follow-up, with no recurrence or new diagnoses, except for one patient. Miscellaneous etiologies accounted for 14.9% of cases, and more than half of these were newly diagnosed primary immunodeficiencies. C-reactive protein and ferritin levels were significantly higher in the inflammatory disease group compared to the groups with unknown and infectious etiologies. In the binary logistic regression analysis, longer fever duration combined with elevated ferritin level was a combined predictor of non-infectious causes (AUC = 0.718). **Conclusions**: Infectious and inflammatory conditions predominate in pediatric FUO, yet a subset of cases resolve spontaneously and follow a benign course. The combination of fever duration and ferritin count may aid early differentiation of non-infectious etiologies, supporting more focused diagnostic approaches. Given the notable proportion of primary immune deficiencies, especially in populations with high consanguinity, early immunologic screening should be incorporated into FUO evaluation protocols.

## 1. Introduction

Fever of unknown origin (FUO) is one of the most intricate diagnostic challenges in childhood. Following Petersdorf and Beeson’s classical definition of FUO in 1961, the description and classification of FUO have evolved. The old definition of fever lasting more than 3 weeks at 38.3 °C or higher in outpatient visits has been shortened in subsequent reports (ranging from 5 days to 3 weeks). In addition, fever in immunocompromised patients was excluded from the new description. In recent pediatric literature, a 7-day definition has been increasingly adopted, aligning with advances in diagnostic techniques that facilitate earlier and more comprehensive evaluation [1,2,3,4,5,6,7]. The term FUO encompasses a heterogeneous group of illnesses rather than a specific diagnosis. Infectious, inflammatory, neoplastic, and miscellaneous reasons can be categorized under this term, while an increasing number of self-limited FUO cases are being reported. Despite advances in diagnostic techniques, including imaging and new microbiological tests, many cases with FUO remain undefined even after a detailed diagnostic work-up. Fever of unknown origin in pediatric populations poses a significant diagnostic challenge in clinical practice, necessitating a comprehensive evaluation to identify the underlying etiologies [8,9]. This diagnostic uncertainty not only prolongs the hospitalization period but also leads to significant anxiety for families [10]. Consequently, clinicians’ strategic selection and prioritization of diagnostic tests constitute a critical component of the diagnostic algorithm for FUO. This study aims to investigate the clinical features and underlying etiologies of FUO, analyze long-term follow-up of children with FUO, and evaluate whether the etiology of FUO in pediatric patients has changed.

## 2. Materials and Methods

This study evaluated children aged 1 month to 18 years who were admitted with a fever of more than 38.3 °C lasting more than one week without an identifiable focus after initial investigations. The study was conducted at İstanbul University-Cerrahpaşa Medical Faculty, Department of Pediatrics, a tertiary-level pediatric referral center, between January 2018 and December 2024. Patients who presented to the outpatient clinic of pediatric infectious diseases or were consulted by the department of pediatric infectious diseases department due to prolonged fever were included. Non-contact infrared devices were used to measure all patients’ body temperatures. Fever duration was defined as the total number of at least seven consecutive febrile days, including both the pre-admission and hospitalization period. Patients with fever lasting more than seven days but with intermittent afebrile days were excluded. Also, children diagnosed with chemotherapy-induced febrile neutropenia and hospital-acquired infections were excluded from the study. All patients were hospitalized and followed in the general pediatric ward. The study was approved by the Istanbul Atlas University Clinical Research Ethics Committee (30.04.2025-64853)

### 2.1. Study Groups and Data Collection

The following data were extracted from hospital electronic medical records as part of the initial evaluation: Age, gender, comorbidities, duration of fever, pathologic physical examination findings, complaints (malaise, rash, abdominal pain, cough, night sweating), laboratory results (complete blood cell count, liver function tests and kidney function tests, C-reactive protein (CRP), erythrocyte sedimentation rate, urinalysis, urine and blood cultures, peripheral blood smear examination. Children who remained undiagnosed despite all work-up and had a fever > 38.3 °C for at least seven consecutive days were defined as having FUO. Children were hospitalized, and second-step diagnostic evaluations were conducted based on the patients’ complaints and physical examination findings. Patients were divided into subgroups as infectious diseases, inflammatory diseases, malignancies, unknown cases, and miscellaneous etiologies. Additionally, a binary classification was applied to compare the infectious disease group and the non-infectious disease group (including inflammatory, malignant, miscellaneous, and unknown etiology cases). Patients were divided into three age groups: <6 years, 6–12 years, and >12 years. The distribution of FUO etiologies among these age groups was compared using the chi-square test. The follow-up examinations for all patient groups were reviewed through the hospital’s electronic system. Patients were diagnosed with hemophagocytic lymphohistiocytosis (HLH) based on the criteria, which require at least five of the following features: fever ≥ 38.5 °C, splenomegaly, cytopenias affecting at least two blood cell lines, hypofibrinogenemia or hypertriglyceridemia, ferritin ≥ 500 μg/L, hemophagocytosis, low or absent NK-cell activity, and elevated sCD25 levels [11].

### 2.2. Microbiological Evaluation

Special microbiological tests (serology or polymerase chain reaction), imaging tests (chest radiography, ultrasonography, computed tomography, echocardiography, magnetic resonance imaging, and positron emission tomography), and pathological examinations (lymph node, bone marrow aspiration) were classified as second-step diagnostic tools. Blood cultures were obtained under sterile conditions and processed using the BACTEC system (Becton Dickinson, Franklin Lakes, NJ, USA). Positive samples were subcultured on appropriate media and incubated under standard aerobic or anaerobic conditions. Microorganisms were identified by Gram staining and colony morphology, and confirmed using MALDI-TOF mass spectrometry. Antimicrobial susceptibility testing was performed using the disc diffusion method and interpreted according to EUCAST guidelines. Cefoxitin discs (30 µg) were used to determine methicillin resistance in Gram-positive cocci, while Gram-negative bacteria were tested with relevant antibiotic discs according to clinical breakpoints. Quality control strains were included in each test run. For the diagnosis of *Mycobacterium tuberculosis* in pediatric patients, samples such as gastric aspirates, sputum, or cerebrospinal fluid were cultured on Löwenstein–Jensen medium and in the MGIT 960 system (Becton Dickinson, USA). Polymerase chain reaction assays were used in selected cases for rapid detection. Tuberculin skin test administered by experienced personnel using the Mantoux method. Indurations ≥ 10 mm were considered positive in at-risk or unvaccinated children, while ≥15 mm was used for low-risk, BCG-vaccinated individuals. All results were interpreted in conjunction with clinical and radiological findings.

### 2.3. Biochemical and Hematological Evaluation

Hematological parameters were analyzed using an automated hematology analyzer (Cell-Dyn 3700, Abbott Company, Abbott Park, IL, USA). Biochemical markers, including C-reactive protein (CRP), procalcitonin, liver function tests, and renal function tests, were measured with a standard clinical chemistry analyzer (Roche/Hitachi, Basel, Switzerland) according to the manufacturer’s protocols.

### 2.4. Statistical Analysis

Descriptive statistical analysis was performed to summarize demographic and clinical variables. Continuous variables were expressed as means with standard deviations or as medians with minimum and maximum values, as appropriate. Categorical variables were reported as frequencies and percentages. The Kolmogorov–Smirnov and Shapiro–Wilk tests were used to assess the normality of distribution. Comparisons between etiological groups were performed for age, laboratory values, and duration of fever using the Kruskal–Wallis test and one-way ANOVA. For post hoc analysis following the Kruskal–Wallis test, pairwise Mann–Whitney U tests were conducted with the Bonferroni correction for multiple comparisons. Subsequently, a binary logistic regression analysis was performed to evaluate the predictive value of clinical parameters in differentiating infectious and non-infectious etiologies of fever. Variables for the regression analysis were selected based on parameters that showed statistical significance in the Mann–Whitney U test comparisons between the infectious and non-infectious groups (Supplementary Table S1). All significant variables were tested individually and in pairwise combinations, and the model with the highest statistical predictive performance was selected as the final model. The model’s performance was assessed using receiver operating characteristic (ROC) analysis. Area under the curve (AUC) values, along with 95% confidence intervals (CI), were reported. Statistical significance was set at *p* < 0.05.

## 3. Results

### 3.1. Patient Characteristics

This study cohort included 87 patients, with a nearly equal gender distribution (54% male, 46% female). The median age was 60 months (IQR: 24.0–120.75 months), and the median duration of fever was 10.0 days (IQR: 8.25–19.25 days). There was no significant difference in the distribution of FUO etiologies among the age groups (<6 years, 6–12 years, and >12 years) (*p* = 0.760). Notably, 98.8% of patients had previously visited another healthcare facility before their admission, and 22.1% had more than one admission to a healthcare facility.

### 3.2. Clinical Presentation

The most frequently reported symptoms beyond fever were rash (31%) and fatigue (27.5%), followed by musculoskeletal pain (25.2%). Respiratory symptoms (cough, chest pain) and gastrointestinal symptoms (nausea/vomiting) were less frequent. Maculopapular rash (31%), hepatomegaly (13.7%), and cervical lymphadenopathy (12.6%) were the most commonly observed pathological physical examination findings. The duration of fever ranged from 7 to over 28 days. Most patients (56%) had a fever lasting 7–13 days, followed by 28.7% with a fever lasting 14–20 days. A total of sixteen (18.6%) patients had comorbid conditions (Table 1).

### 3.3. Etiologies

Infectious and inflammatory diseases were the most common etiologies (42.5% and 19.5%, respectively), followed by the unknown group, malignancies, and other miscellaneous diseases (Table 1). The median age and sex distribution did not differ significantly among the groups (*p* = 0.72, *p* = 0.114, respectively). In the undefined group, the fever resolved spontaneously, and no new complaints were observed during outpatient follow-up, except for one patient who was lost to follow-up. The etiological causes of prolonged fever in all patients are comprehensively summarized in Table 2.

### 3.4. Laboratory Findings and Group Comparisons

According to the Kruskal–Wallis test (Table 3), statistically significant differences were observed among the five groups regarding fever duration (*p* = 0.026), C-reactive protein levels (*p* = 0.003), sedimentation rate (*p* = 0.001), neutrophil count (*p* = 0.038), hemoglobin level (*p* = 0.009), platelet count (*p* = 0.044), and ferritin levels (*p* = 0.00). Post hoc analysis using the Mann–Whitney U test and the Bonferroni correction (*p* < 0.005) revealed that CRP and ferritin levels were significantly higher in the inflammatory disease group compared to the unknown and infectious etiology groups (Table 4). Additionally, the sedimentation rate was significantly higher in the inflammatory disease group compared to the unknown group (*p* = 0.002). According to the Mann–Whitney U test, several laboratory parameters were significantly different between the non-infectious group and the infectious group. Patients in the non-infectious group had a longer duration of fever (*p* = 0.003) and higher ferritin levels (*p* = 0.09). Additionally, the white blood cell count (*p* = 0.024), neutrophil count (*p* = 0.004), platelet count (*p* = 0.015), and ferritin levels (*p* = 0.004) were significantly higher in the non-infectious group. Based on clinical relevance and statistical significance, a binary logistic regression model was constructed using fever day and ferritin to differentiate infectious and non-infectious causes. The model was statistically significant (Chi-square = 16.998, *p* < 0.001), explaining 23.8% of the variance (Nagelkerke R^2^ = 0.238) and correctly classifying 64.4% of cases (Table 5). ROC curve analysis demonstrated an area under the curve (AUC) of 0.718 (95% CI: 0.612–0.824, *p* = 0.001).

## 4. Discussion

Fever of unknown origin in children represents a significant clinical challenge, often necessitating an extensive diagnostic work-up to identify the underlying cause. This study summarizes the leading causes of FUO in pediatric patients at our hospital. The median age in our study (60 months) was lower compared to other pediatric FUO reports. In many studies investigating the etiology of FUO in children, infectious diseases have been reported as the most common cause [6,12,13]. Similarly, in our study, infectious diseases (44,1%) were the most frequent cause, followed by inflammatory diseases and the undefined group. The median fever duration in our study was 10 days. In our cohort, 56% of patients experienced a fever lasting 7–13 days, whereas only 11.4% had a fever lasting more than 28 days. In contrast, Kim et al. reported that 46% of patients diagnosed with fever of unknown origin had a fever duration exceeding 28 days [12]. This discrepancy may be attributable to variations in the study population or the spectrum of underlying diseases.

Regarding the presenting complaints of patients, rash often constitutes an early clinical sign of an underlying etiology, including infectious and inflammatory disorders. In our series, rash was detected in 31% of patients, a frequency similar to that reported by Shoman et al. (24.5%) and Demir et al. (30.7%) [14,15]. This consistency across studies emphasizes the diagnostic importance of cutaneous manifestations in pediatric FUO.

In our study, when comparing laboratory results across different etiology groups, we found that CRP and ferritin levels were significantly higher in the inflammatory disease group compared to the unknown and infectious etiology groups in pairwise comparisons (Table 4). In contrast, Antoon et al. [6] did not observe a significant difference in CRP or ferritin levels among the various etiology groups; however, they reported higher sedimentation rates in both the infection and autoimmune disease groups. The higher CRP and ferritin levels observed in the inflammatory group may be attributed to the chronic and systemic nature of immune-mediated inflammation, as well as possible delays in diagnosis, which could lead to further elevation of inflammatory markers.

The combined presence of prolonged fever and elevated ferritin levels was found to be significantly associated with non-infectious etiologies in our cohort, as demonstrated by our binary logistic regression analysis (AUC: 0.718). While ferritin was elevated in inflammatory cases, it did not perform well as an independent threshold for FUO. Its value became clear only when combined with fever duration; therefore, we did not propose a single cut-off for ferritin and interpreted it as a supportive marker in conjunction with fever duration. In a recent retrospective study by Rienvichit et al. [5], a predictive model developed for pediatric FUO cases demonstrated that fever duration exceeding 30 days was significantly associated with autoimmune diseases and malignancies. These findings support the notion that the likelihood of non-infectious etiologies increases as the duration of fever prolongs. Ferritin, beyond being an acute-phase reactant, is a reliable indicator of immune activation, particularly in disorders such as systemic juvenile idiopathic arthritis and hemophagocytic lymphohistiocytosis. While ferritin was not included in the predictive model proposed by Rienvichit et al., we demonstrated that its elevation, along with prolonged fever, independently predicts non-infectious causes in pediatric FUO.

In numerous studies conducted over different years, infectious diseases have remained the leading cause of FUO in children. There are some differences in the etiological factors between developed and developing countries. For instance, the prevalence of Bartonella and Epstein–Barr virus is higher in developed countries, whereas typhoid fever and tuberculosis are more frequently observed in non-developed countries [8,9]. In our study, viruses were the most common pathogens, followed by bacterial agents, in terms of infectious etiology. Adenovirus was the most frequently detected virus (9%) causing FUO, followed by EBV and human herpesvirus 6. L’Huillier et al. [16] reported that 34.8% of children with fever without a source had a positive polymerase chain reaction (PCR) for at least one virus, underscoring the importance of point-of-care viral tests in reducing the need for detailed laboratory workups. Although we did not have the opportunity to test blood for viruses using real-time PCR in our study, we were able to investigate viral infections in all patients using a multiplex PCR upper respiratory tract panel and serologic tests. Bacterial infections, particularly those caused by *Brucella, Bartonella henselae*, and *Mycobacterium tuberculosis*, were the second most frequently identified causes among infectious agents in our cohort. Unlike our findings, *Rickettsia rickettsii*, *Borrelia burgdorferi, Coxiella burnetti*, and *Leptospira* spp. have been identified as the most common bacterial pathogens associated with the etiology of FUO in various endemic regions [17].

In our country, where tuberculosis has moderate endemicity, it was identified in three patients, one of whom was concurrently diagnosed with chronic granulomatous disease (CGD). Primary immune deficiencies, such as chronic granulomatous disease (CGD) or defects in the interferon-gamma and interleukin-12 pathways, are significant contributors to susceptibility to mycobacterial infections. Therefore, screening for immune deficiencies in children with tuberculosis would be a logical approach. Another endemic pathogen, *Brucella*, was identified in four patients, all of whom had either originated from the eastern regions of the country, had a family history of livestock farming, or reported consuming fresh cheese. Similarly, Çiftçi et al. [18] reported Brucellosis as a common cause of FUO in children. In addition to Brucella, Bartonella henselae is another infectious cause of FUO, commonly associated with suppurative lymphadenopathy, as also highlighted by Öcal et al. [19]. However, the two patients diagnosed with cat scratch disease in our study presented with prolonged fever and systemic organ involvement—with hypoechoic nodules in the liver and spleen, accompanied by bone involvement—without accompanying lymphadenopathy. Similarly, Tsujino et al. [20] found an association between the absence of lymphadenopathy and prolonged fever in their study of 127 pediatric patients diagnosed with bartonellosis. They recommended considering cat scratch disease in cases presenting solely with prolonged fever, even in the absence of lymphadenopathy, highlighting that Bartonellosis can progress beyond lymph node involvement to cause systemic manifestations, posing significant diagnostic challenges.

As a major referral center, inflammatory diseases were the second most common cause of prolonged fever, with systemic juvenile idiopathic arthritis (sJIA) and Kawasaki disease being the most frequent diagnoses (8%, 5.7%). Notably, sJIA is a diagnosis of exclusion, typically established only after infectious and malignant causes of fever are ruled out, underscoring the importance of a thorough differential diagnosis. Additionally, vasculitis should be considered in the evaluation of fever of unknown origin (FUO); one of our cases was diagnosed with Takayasu arteritis, a rare large-vessel vasculitis affecting the aorta and its branches. Supporting this, Şahin et al. [21] reported fever as a presenting symptom in 7 out of 16 pediatric patients with Takayasu arteritis, further highlighting its relevance in the FUO spectrum. In addition, two patients in our study were diagnosed with familial Mediterranean fever, a hereditary autoinflammatory disease characterized by recurrent febrile episodes and serositis [22].

Another critical condition to consider in the differential diagnosis of FUO is hemophagocytic lymphohistiocytosis (HLH), characterized by cytopenia, markedly elevated ferritin, and acute-phase reactants, along with a low erythrocyte sedimentation rate [23]. In our cohort, secondary HLH was identified in six patients (6.9%) and was associated with a range of underlying conditions, including lymphoma, immunodeficiency, *Staphylococcus aureus* sepsis, systemic juvenile idiopathic arthritis, and rubella infection, highlighting the heterogeneity of HLH triggers in pediatric populations. On the other hand, primary HLH was identified in two patients among the various underlying causes, as summarized in Table 2. Early laboratory assessment—particularly ferritin, ESR, and peripheral blood smear—is essential for timely recognition. Prompt diagnosis and treatment are crucial to reduce the high risk of mortality associated with HLH.

In our study, the unknown group was identified as the third most common cause, accounting for 17%. A total of 14 patients with spontaneously resolving fever without a definitive diagnosis showed no new complaints or recurrence of fever during outpatient follow-up after discharge. There is scarce data about the long-term follow-up of undefined FUO cases. Statler et al. [24] reported that 69% of children with FUO had no specific diagnosis despite detailed investigations. Also, they emphasized that most of them are self-limited. Weakley et al. [25] reported that only 3 out of 120 FUO patients without an identified etiology were later diagnosed with juvenile idiopathic arthritis (JIA), suggesting that FUO is often a benign, self-limiting condition in the absence of severe disease indicators. In our study, the median outpatient follow-up duration for these children was 63 months (range, 8–90 months). Notably, none of these patients experienced a recurrence of prolonged, unexplained fever during the follow-up period. Furthermore, no definitive diagnosis explaining the initial febrile episode was established in any patient, either in our center or through evaluations conducted at external healthcare institutions. This may be attributed to transient viral infections that go undetected or to mild autoinflammatory conditions presenting as a single, self-limited febrile episode, particularly in younger children.

In our cohort, malignancies and miscellaneous conditions represented the least common causes of FUO. Among miscellaneous etiologies, we identified cases of primary immunodeficiencies (n = 7), inflammatory bowel disease (n = 1), metabolic disorders (n = 1), and Kikuchi-Fujimoto disease (n = 1). The Kikuchi-Fujimoto case was a 5-year-old boy with a 40-day history of fever, prolonged fever, rash, and cervical lymphadenopathy, diagnosed via lymph node biopsy. This rare, benign, self-limiting condition—characterized by histiocytic necrotizing lymphadenitis—has been previously associated with prolonged fever durations, with reported medians around 22 days (IQR: 17–33 days) [26]. Primary immunodeficiencies (PIDs) are increasingly recognized as important yet often undefined causes of fever of unknown origin (FUO). In our study, 33 patients (38.3%) underwent PID screening, and 7 (21%) were ultimately diagnosed with a confirmed immunodeficiency. The literature includes several case reports of children presenting with prolonged fever who were later diagnosed with various PIDs, such as deficiency of adenosine deaminase 2, severe combined immunodeficiency, Wiskott-Aldrich syndrome, and Schimke immunoosseous dysplasia [27,28,29]. In our cohort, Chronic Granulomatous Disease (CGD) was the most frequently identified PID, accounting for four of the seven cases. Among the remaining patients, two were diagnosed with immune dysregulation syndromes and one with a primary antibody deficiency. Notably, three CGD patients had concurrent infectious complications, including tuberculosis, *Salmonella* sepsis, and intra-abdominal abscesses, whereas one patient presented with isolated prolonged fever accompanied by growth failure. Given the higher prevalence of consanguineous marriages in our country, which increases the risk of autosomal recessive diseases, early and comprehensive PID screening is crucial in the evaluation of FUO. Timely diagnosis not only facilitates appropriate management but also helps prevent recurrent or severe infections.

This study has both strengths and limitations. This is a retrospective, single-center study with a relatively small number of patients in less common etiologic subgroups, such as malignancies and miscellaneous conditions. This may have limited the statistical power of subgroup analyses. As ferritin alone had poor performance in regression analysis and improved only in conjunction with fever duration, no single cut-off was established. Future work should focus on validating the multimarker strategy. Another limitation of our study is that we defined FUO as fever lasting seven days or longer, in line with recent pediatric literature. Using longer thresholds, such as 14 or 21 days, might have altered the etiologic distribution; however, these stricter definitions would markedly restrict patient diversity and exclude cases currently considered as prolonged fever in modern clinical practice.

Nevertheless, the study benefits from a relatively large and clinically diverse pediatric cohort evaluated over a multi-year period, as well as a systematic etiologic classification of FUO cases. The inclusion of long-term follow-up data from patients with undefined conditions provides valuable insights. Moreover, multivariable analysis showed that prolonged fever combined with elevated ferritin levels may be particularly useful for distinguishing non-infectious causes of prolonged fever.

## 5. Conclusions

In conclusion, infectious diseases were the most common cause of prolonged fever in children, while inflammatory conditions and undefined cases also contributed. A notable proportion of primary immunodeficiencies highlights the need for early immunologic evaluation, especially in populations with high consanguinity. Clinicians should remain alert for severe complications such as hemophagocytic lymphohistiocytosis in the presence of cytopenia and markedly elevated inflammatory markers. Finally, the combination of prolonged fever duration and elevated ferritin levels may help identify non-infectious etiologies, providing a simple tool for guiding diagnostic strategies.

## Figures and Tables

**Table 1:** Baseline clinical and demographic features of patients with prolonged fever.

		N (%)
Gender	Male	47 (54%)
	Female	40 (46%)
Age	>6 years	54 (61.6%)
	6–12 years	15 (17.4%)
	>12 years	18 (21%)
Duration of fever	7–13 days	49 (56%)
	14–20 days	25 (28.7%)
	21–27 days	3 (3.4%)
	>28 days	10 (11.4%)
Complaints	Rash	27 (31%)
	Fatigue	24 (27.5%)
	Muscle/joint pain	22 (25.2%)
	Cough	14 (16%)
	Weight loss	13 (14.9%)
	Abdominal pain	9 (10.3%)
	Night sweats	8 (9.1%)
	Nausea-vomiting	6 (6.8%)
	Sputum production	6 (6.8%)
	Chest pain	4 (4.6%)
Physical Examination	Maculopapular rash	27 (31%)
	Hepatomegaly	12 (13.7%)
	Cervical lymphadenopathy	11 (12.6%)
	Oropharyngeal findings	8 (9.1%)
	Splenomegaly	7 (8%)
	Generalized lymphadenopathy	4 (4.6%)
	Respiratory findings	2 (2.2%)
	Cardiac murmur	2 (2.2%)
Etiology	Infectious	37 (42.5%)
	Inflammatory	17 (19.5%)
	Unknown origin	15 (17.2%)
	Miscellaneous	13 (14.9%)
	Malignancy	5 (5.9%)
Treatment	Antibiotics	31 (35.6%)
	Corticosteroid	16 (18.3%)
	Biological agent	8 (9.1%)
	Intravenous immune globulin	6 (6.8%)

Oropharyngeal findings consisted of sore throat and oral ulcers, and respiratory findings included cough and dyspnea.

**Table 2:** Categorization of diagnoses based on underlying etiology in children with FUO.

Infectious	Adenovirus (8)
	Brucellosis (5)
	Epstein- Barr Virus (5)
	Tuberculosis (4)
	Bartonellosis (2)
	*Streptococcus pyogenes* (2)
	Human herpesvirus 6 (1)
	Dural abscesses (1)
	*Staphylococcus aureus* (1)
	Cytomegalovirus (1)
Inflammatory	Systemic juvenile idiopathic arthritis (7)
	Kawasaki disease (5)
	Familial Mediterranean fever (2)
	Acute rheumatic fever (1)
	Takayasu arteritis (1)
Miscellaneous	Chronic granulomatous disease (4)
	Primary antibody deficiency (1)
	Immune dysregulation (2)
	Inflammatory bowel disease (1)
	Kikuchi Fujimoto disease (1)
	Propionic acidemia (1)
	Primary hemophagocytic lymphohistiocytosis (2)
Malignancy	Neuroblastoma (2)
	Acute lymphoblastic leukemia (1)
	Myelodysplastic syndrome (1)
	NK cell lymphoma (1)

**Table 3:** Comparative analysis of laboratory findings by etiological category median (minimum-maximum) or mean (±standard deviation).

	Infectious	Inflammatory	Unknown	Miscellaneous	Malignancy	*p*
Age (month)	58 (10–192)	72 (5–189)	33 (6–193)	60 (3–204)	67.5 (48–109)	0.72 *
Fever duration (day)	10 (5–30)	14 (8–30)	14 (7–50)	11 (9–40)	14.5 (10–40)	0.026 *
C-Reactive protein (mg/L)	23 (0.6–216)	107 (13–333)	15.4 (1–165)	25 (3–255)	43.5 (5.5–79)	0.003 *
Sedimentation rate (mm/h)	30 (2–475)	79 (27–124)	13 (2–109)	61 (3–91)	63 (17–99)	0.001 *
Hemoglobin (g/dL)	10.6 (8–15)	9.5 (8–12)	11.3 (7.1–14.5)	8.3 (8–13)	9.65 (8–10)	0.009 *
Total leucocyte count (/µL)	9500 (1300–22,000)	13,900 (2200–37,600)	11,600 (3970–23,300)	12,400 (4400–29,400)	10,545 (4600–220,400)	0.206 *
Neutrophil count (/µL)	4000 (840–15,900)	8400 (400–34,800)	7500 (1500–15,500)	6400 (600–18,500)	5505 (2300–102,900)	0.038 *
Lymphocyte count (/µL)	3000 (600–13,600)	2100 (640–8000)	4000 (800–6300)	3790 (600–13,200)	3300 (1300–42,600)	0.94 *
Platelet count (/µL)	286,000 (18,200–761,000)	509,000 (38,000–1,000,000)	449,000 (159,000–853,000)	332,000 (13,000–613,000)	312,500 (35,000–833,000)	0.044 *
Ferritin (ng/mL)	142 (15–2120)	750 (6–25.400)	120 (38–387)	286 (25–2943)	254.5 (15–745)	0.00 *
Aspartate aminotransferase IU/L	58.9 ± 80.4	41.8 ± 32.4	28.3 ± 10.09	50.5 ± 53.8	43 ± 19.7	0.107 ^&^
Alanine aminotransferase IU/L	63.7 ± 134.9	36.7 ± 39.6	14.7 ± 8.2	29.2 ± 22.4	18.5 ± 8.7	0.035 ^&^
Lactate dehydrogenase IU/L	302 (178–825)	289 (133–623)	323 (171–387)	361 (187–662)	444 (192–1125)	0.496 *

* Kruskal–Wallis test, ^&^ One-way ANOVA test. Measurement units: C-reactive protein–milligrams per liter (mg/L); Erythrocyte sedimentation rate–millimeters per hour (mm/h); Hemoglobin–grams per liter (g/L); Total leucocyte count-cells per microliter; Neutrophil count-cells per microliter; Lymphocyte count-cells per microliter; Platelet count-cells per microliter; Ferritin–nanograms per milliliter (ng/mL); Aspartate aminotransferase-international units per liter (IU/L); Alanine aminotransferase-international units per liter (IU/L); Lactate dehydrogenase-international units per liter (IU/L).

**Table 4:** Statistically significant pairwise comparisons (Mann–Whitney U test with Bonferroni correction) of laboratory parameters between diagnostic groups (*p* < 0.005).

	Fever Day	Sedimentation	Ferritin	CRP	Neutrophil	Hemoglobin	Platelet
Inflammatory-Malignancy	0.323	0.319	0.074	0.050	0.327	0.889	0.327
Inflammatory-Infection	0.108	0.038	0.000	0.001	0.002	0.010	0.017
Inflammatory-Miscellaneous	0.220	0.018	0.263	0.066	0.232	0.982	0.132
Inflammatory-Unknown	0.760	0.002	0.000	0.000	0.331	0.005	0.865
Malignancy-Infection	0.024	0.083	0.149	0.888	0.344	0.049	0.861
Malignancy-Miscellaneous	0.635	0.494	0.616	0.750	0.750	0.820	0.892
Malignancy-Unknown	0.507	0.533	0.094	0.243	0.846	0.011	0.302
Infection-Miscellaneous	0.009	0.226	0.018	0.5	0.137	0.059	0.907
Infection-Unknown	0.072	0.454	0.443	0.096	0.120	0.492	0.011
Miscellaneous-Unknown	0.540	0.241	0.014	0.124	0.961	0.092	0.075

**Table 5:** Binary logistic regression analysis for predicting non-infectious etiology.

		B	S.E.	Wald	df	Sig.	Exp(B)	95% C.I. for EXP(B)	
								Lower	Upper
Step 1 ^a^	feverday	−0.090	0.039	5.301	1	0.021	0.914	0.846	0.987
	ferritin	−0.001	0.001	3.175	1	0.075	0.999	0.998	1.000
	Constant	1.334	0.575	5.376	1	0.020	3.797		

a. Variables entered in the logistic regression model: fever duration and ferritin.

## Data Availability

The data supporting the findings of this study are not publicly available due to privacy restrictions.

## Supplementary Materials

The following supporting information can be downloaded at: https://www.mdpi.com/article/10.3390/children12111493/s1, Table S1: Comparison of Clinical and Laboratory Findings Between Infectious and Non-infectious Etiologies.

## Abbreviations

The following abbreviations are used in this manuscript:

FUO	Fever of unknown origin
CRP	C-reactive protein
ROC	Receiver operating characteristic
AUC	Area under the curve
CI	Confidence intervals
EBV	Epstein–Barr virus
PCR	Polymerase chain reaction
CGD	Chronic granulomatous disease
JIA	Juvenile idiopathic arthritis
HLH	Hemophagocytic lymphohistiocytosis
PID	Primary immune deficiency

## References

[1] Petersdorf R.G., Beeson P.B. (1961). Fever of unexplained origin: Report on 100 cases. Medicine.

[2] Durack D.T., Street A.C. (1991). Fever of unknown origin-reexamined and redefined. Curr. Clin. Top. Infect. Dis..

[3] Dhangar P., Panda P.K., Kant R., Gupta R., Dua R., Tiwari A., Saini S., Khoiwal K., Bahurupi Y. (2025). Evaluating fever of unknown origin definitions in a tertiary care setting: Implications for diagnostic criteria revision. World J. Exp. Med..

[4] Wright W.F., Auwaerter P.G. (2020). Fever and Fever of Unknown Origin: Review, Recent Advances, and Lingering Dogma. Open Forum Infect. Dis..

[5] Rienvichit P., Lerkvaleekul B., Apiwattanakul N., Pakakasama S., Rattanasiri S., Vilaiyuk S. (2025). Prediction model for etiology of fever of unknown origin in children. Eur. J. Pediatr..

[6] Antoon J.W., Peritz D.C., Parsons M.R., Skinner A.C., Lohr J.A. (2018). Etiology and Resource Use of Fever of Unknown Origin in Hospitalized Children. Hosp. Pediatr..

[7] Bouhmidi M., Babakhouya A., El Ouali A., Ghannam A., Rkain M., Benajiba N. (2021). P074 Prolonged fever in children: Epidemiological and etiological profile in the eastern region of Morocco—About 119 cases. Rheumatology.

[8] Trapani S., Fiordelisi A., Stinco M., Resti M. (2023). Update on Fever of Unknown Origin in Children: Focus on Etiologies and Clinical Approach. Children.

[9] Haidar G., Singh N. (2022). Fever of Unknown Origin. N. Engl. J. Med..

[10] Çelik T., Güzel Y. (2024). Parents’ Knowledge and Management of Fever: “Parents versus Fever!”. Turk. Arch. Pediatr..

[11] Henter J.I. (2025). Hemophagocytic Lymphohistiocytosis. N. Engl. J. Med..

[12] Kim Y.S., Kim K.R., Kang J.M., Kim J.M., Kim Y.J. (2017). Etiology and clinical characteristics of fever of unknown origin in children: A 15-year experience in a single center. Korean J. Pediatr..

[13] Hu B., Chen T.M., Liu S.P., Hu H.L., Guo L.Y., Chen H.Y., Li S.Y., Liu G. (2022). Fever of unknown origin (FUO) in children: A single-center experience from Beijing, China. BMJ Open..

[14] Shoman W., Galal A., Elshishiny A.M., Hamza E. (2024). Fever of unknown origin in pediatrics: Causes and clinical characteristics in a single centre experience. Gaz. Egypt. Pediatr. Assoc..

[15] Demir O., Kılıç M.Ç., Kışla R.E., Çelik Ü. (2024). Fever of Unknown Origin in Children: A Single Center Experience from Southern Türkiye. J. Pediatr. Inf..

[16] L’Huillier A.G., Mardegan C., Cordey S., Luterbacher F., Papis S., Hugon F., Kaiser L., Gervais A., Posfay-Barbe K., Galetto-Lacour A. (2020). Enterovirus, parechovirus, adenovirus, and herpes virus type 6 viremia in fever without source. Arch. Dis. Child..

[17] Erdem H., Baymakova M., Alkan S., Letaief A., Yahia W.B., Dayyab F., Kolovani E., Grgic S., Cosentino F., Hasanoglu I. (2023). Classical fever of unknown origin in 21 countries with different economic development: An international ID-IRI study. Eur. J. Clin. Microbiol. Infect. Dis..

[18] Ciftçi E., Ince E., Doğru U. (2003). Pyrexia of unknown origin in children: A review of 102 patients from Turkey. Ann. Trop. Paediatr..

[19] Öcal-Demir S., Kahraman K., Bozbeyoğlu G., Esen F. (2024). Clinical Presentation of Cat Scratch Disease in Pediatric Patients—A Single-Center Study. Turk. Arch. Pediatr..

[20] Tsujino K., Tsukahara M., Tsuneoka H., Ichihara K., Furuya T., Kawauchi S., Oga A., Sasaki K. (2004). Clinical implication of prolonged fever in children with cat scratch disease. J. Infect. Chemother..

[21] Sahin S., Hopurcuoglu D., Bektas S., Belhan E., Adrovic A., Barut K., Canpolat N., Caliskan S., Sever L., Kasapcopur O. (2019). Childhood-onset Takayasu arteritis: A 15-year experience from a tertiary referral center. Int. J. Rheum. Dis..

[22] Kisla Ekinci R.M., Kilic Konte E., Akay N., Gul U. (2024). Familial Mediterranean Fever in Childhood. Turk. Arch. Pediatr..

[23] Baldo F., Erkens R.G.A., Mizuta M., Rogani G., Lucioni F., Bracaglia C., Foell D., Gattorno M., Jelusic M., Anton J. (2025). PReS MAS/sJIA Working Party and Paediatric Rheumatology International Trial Organization. Current treatment in macrophage activation syndrome worldwide: A systematic literature review to inform the METAPHOR project. Rheumatology.

[24] Statler V.A., Marshall G.S. (2016). Characteristics of Patients Referred to a Pediatric Infectious Diseases Clinic with Unexplained Fever. J. Pediatric Infect. Dis. Soc..

[25] Weakley K.E., Marshall G.S., Statler V.A. (2020). Long-term Health Outcomes of Patients Evaluated for Unexplained Fever in a Pediatric Infectious Diseases Clinic. J. Pediatric Infect. Dis. Soc..

[26] Lou D., Song Y. (2024). Clinical features of histiocytic necrotizing lymphadenitis in children. Eur. J. Pediatr..

[27] Nihira H., Nakagawa K., Izawa K., Kawai T., Yasumi T., Nishikomori R., Nambu M., Miyagawa-Hayashino A., Nomura T., Kabashima K. (2018). Fever of unknown origin with rashes in early infancy is indicative of adenosine deaminase type 2 deficiency. Scand. J. Rheumatol..

[28] Jamee M., Khakbazan Fard N., Fallah S., Golchehre Z., Fallahi M., Shamsian B.S., Sharafian S., Chavoshzadeh Z. (2022). Cernunnos defect in an Iranian patient with T-B^+^NK^+^ severe combined immunodeficiency: A case report and review of the literature. Mol. Genet. Genom. Med..

[29] Taha D., Boerkoel C.F., Balfe J.W., Khalifah M., Sloan E.A., Barbar M., Haider A., Kanaan H. (2004). Fatal lymphoproliferative disorder in a child with Schimke immuno-osseous dysplasia. Am. J. Med. Genet. A.

