# Supplementary material for: Diagnostic Challenges in Pediatric Fever of Unknown Origin: Combined Role of Ferritin and Fever Duration
Source: Children (Basel). 2025 Nov 4;12(11):1493. doi: 10.3390/children12111493 (PMC12651891; doi:10.3390/children12111493)
Supplement: Supplementary file 1 [file children-12-01493-s001.zip › children-3943242-supplementary.pdf]

Supplementary Table S1: Table: Comparison of Clinical and Laboratory Findings Between Infectious and Non-infectious Etiologies (Median [Min–Max], Mann–Whitney U Test)

	Infectious	Non-infectious	*p*
Age (months)	58 (10-192)	70,3 (3-204)	0,453
Fever duration (day)	10 (7-30)	14 (7-50)	**0,004**
Total leucocyte count (/mm3)	9500 (1300-22000)	11600 (2200-224000)	**0,017**
Neutrophil count (/mm3)	4000 (840-15900)	8000 (400-102900)	**0,003**
Lymphocyte count (/mm3)	3000(600-13600)	3500(600-42600)	0,896
Hemoglobin (g/dl)	10,6 (8-14,6)	10 (7,1-14,5)	0,051
Platelet count (/mm3)	286000 (18200-761000)	405000 (13000-	**0,019**
Ferritin (ng/mL)	151(15-1100)	242(6-25400)	**0,011**
Lactat dehydrogenase (IU/lt)	302(178-825)	324 (133-1125)	0,886
Aspartate aminotransferase (IU/lt)	33 (14-404)	29 (7-200)	0,324
Alanine aminotransferase (IU/lt)	21 (4-613)	17(4-147)	0,301
C-reactive protein (mg/lt)	23(0-216)	40,1(1-333)	0,331
Sedimentation rate (mm/h)	48 (2-475)	61(2-124)	0,079
